# The impact of perceived organizational support on nurses' safety behavior: the sequential mediating roles of decent work perception and job embeddedness

**DOI:** 10.3389/fpubh.2026.1868271

**Published:** 2026-07-28

**Authors:** Huihua Tang, Qianming Xie, Chunyan Liao, Guowei Chen, Lili Zhang, Meifang Mo, Hangrui Zhang

**Affiliations:** 1 College of Nursing, Guilin Medical University, Guilin, China; 2 The First Affiliated Hospital of Guilin Medical University, Guilin, China

**Keywords:** chain mediation effect, decent work perception, job embeddedness, nurses, nurses' safety behavior, perceived organizational support

## Abstract

**Objective:**

To examine the sequential mediating role of decent work perception and job embeddedness of decent work perception and job embeddedness in the relationship between perceived organizational support and nurses' safety behavior.

**Methods:**

A multicenter cross-sectional survey was conducted among 753 nurses from four tertiary grade-A hospitals in Guangxi Zhuang Autonomous Region, China. Data were collected using a demographic questionnaire and validated scales measuring perceived organizational support, decent work perception, job embeddedness, and nurses' safety behavior. Spearman's rank correlation analysis and structural equation modeling with bootstrap resampling were conducted.

**Results:**

Mean scores for perceived organizational support, decent work perception, job embeddedness, and nurses' safety behavior were 40.92 ± 9.86, 55.99 ± 7.59, 65.30 ± 11.08, and 55.28 ± 4.22, respectively. Perceived organizational support, decent work perception, and job embeddedness were positively correlated with nurses' safety behavior. The total indirect effect of perceived organizational support on nurses' safety behavior was 0.297, accounting for 51.83% of the total. The indirect effects through decent work perception and job embeddedness accounted for 14.14% and 23.90% of the total effect, respectively, and the sequential indirect effect through decent work perception and job embeddedness accounted for 13.79%.

**Conclusions:**

Decent work perception and job embeddedness mediated the association between perceived organizational support and nurses' safety behavior. Strengthening organizational support, decent work perception, and job embeddedness may help promote safer nursing practice.

## 1 Introduction

Patient safety is a central concern in healthcare systems worldwide (1). Nurses' safety behavior is essential for improving patient safety and reducing adverse events (2). In China, nurses face multiple challenges, including heavy workloads, shift-related fatigue, and increasingly complex nurse–patient relationships, all of which may influence their safety behavior (3). Inadequate safety behavior may compromise patient safety and care quality (4). Perceived organizational support has been identified as an important predictor of nurses' safety behavior (5). It refers to employees' perception of the extent to which the organization values their contributions and cares about their wellbeing (6). Evidence suggests that higher perceived organizational support is associated with greater compliance with safety procedures and more active engagement in safety-related activities (7). However, the mechanisms through which perceived organizational support influences nurses' safety behavior remain insufficiently understood, and the potential psychological pathways warrant further investigation.

Social exchange theory provides a useful framework for understanding this mechanism (8). It suggests that workplace behavior is shaped by reciprocal exchanges between employees and organizations. When employees receive valuable resources, support, and recognition from their organization, they tend to feel an obligation to respond with positive attitudes and behaviors that benefit the organization. In the context of nurses' safety behavior, perceived organizational support represents a key organizational resource that may trigger a series of psychological processes (9). Decent work perception refers to employees' perception that their work is carried out under conditions of dignity, fairness, security, and respect, with emphasis on personal value and recognition (10). In high-pressure clinical environments, decent work perception may help nurses maintain professional meaning, reduce psychological strain related to lack of recognition, and enhance motivation to engage in safe clinical practice. Previous studies have also reported a positive association between decent work perception and safety-related behaviors (11). Therefore, this study proposes the following hypothesis: H1: Decent work perception mediates the relationship between perceived organizational support and nurses' safety behavior.

Job embeddedness refers to the extent to which employees are connected to their job and organization, including links, fit, and perceived sacrifices associated with leaving (12). Higher job embeddedness strengthens nurses' psychological attachment and organizational identification, reduces disengagement, and promotes greater commitment to safe and responsible clinical practice (13). Accordingly, nurses with higher job embeddedness are more likely to maintain safe behaviors in clinical settings. Therefore, this study proposes the following hypothesis: H2: Job embeddedness mediates the relationship between perceived organizational support and nurses' safety behavior.

In addition to their independent mediating effects, decent work perception and job embeddedness may function in a sequential manner. Perceived organizational support reflects the resources, care, and recognition provided by the organization. Such supportive experiences may enhance nurses' perceptions that their work is meaningful, respected, and valuable, thereby strengthening their decent work perception. Decent work perception represents nurses' cognitive and emotional evaluation of the dignity and value of their work, which may further foster stronger attachment to their job, colleagues, and organization, thereby enhancing job embeddedness. In this sense, decent work perception reflects an evaluative perception of work meaning and dignity, whereas job embeddedness reflects a more stable form of organizational attachment and integration. This conceptual distinction provides a theoretical basis for the proposed directional pathway from decent work perception to job embeddedness. Consistent with prior evidence on sequential mediation mechanisms in occupational behavior research (14), perceived organizational support may enhance nurses' decent work perception, which in turn strengthens job embeddedness and ultimately promotes nurses' safety behavior. Therefore, this study proposes the following hypothesis: H3: Decent work perception and job embeddedness sequentially mediate the relationship between perceived organizational support and nurses' safety behavior.

At present, limited empirical evidence has clarified how perceived organizational support influences nurses' safety behavior through the sequential pathway of decent work perception and job embeddedness. To address this gap, this study applies social exchange theory to examine the association between perceived organizational support and nurses' safety behavior and to test the sequential mediating roles of decent work perception and job embeddedness. By elucidating this sequential mechanism, the findings may offer a more comprehensive understanding of how organizational resources are translated into safety-related behaviors. This may provide a theoretical basis for developing targeted strategies to improve nurses' safety behavior, including strengthening organizational support, enhancing decent work perception, and promoting job embeddedness, ultimately contributing to improved nursing quality and patient safety.

## 2 Methods

### 2.1 Design

This study employed a multicenter cross-sectional survey design to examine the relationships among perceived organizational support, decent work perception, job embeddedness, and nurses' safety behavior.

### 2.2 Participants

Participants were recruited from four tertiary grade-A hospitals in Guangxi Zhuang Autonomous Region, China, between October and December 2024 using convenience sampling. Eligible participants were clinical nurses who (1) held a valid nursing qualification certificate, (2) were engaged in direct clinical nursing practice during the study period, and (3) provided informed consent to participate. Nurses were excluded if they were not engaged in clinical nursing practice (e.g., administrative nurses), were absent during the survey period due to maternity leave, sick leave, study leave, or other reasons, or were undergoing standardized training, departmental rotation, internship, probation, or post-retirement reemployment.

The required sample size was estimated based on Kendall's principle, which suggests a minimum of 10 participants per variable (15). Given 17 variables (eight demographic variables and nine scale-based variables), the minimum required size was 170. After accounting for a 20% potential invalid response rate, the target sample size was set at 204. To ensure adequate statistical power for structural equation modeling and stable parameter estimation, a larger sample size was adopted. A total of 753 valid responses were finally included, exceeding the minimum requirement and meeting the requirements for the proposed mediation analysis.

### 2.3 Measures

#### 2.3.1 The demographic characteristics questionnaire

The demographic questionnaire was developed by the research team based on a review of previous studies (16–18) and the clinical relevance of demographic and professional characteristics to nurses' safety behavior. It was used to collect participants' background information, including sex, age, educational level, marital status, professional title, years of work experience, teaching responsibilities, and participation in standardized training.

#### 2.3.2 The Perceived Organizational Support Scale (POSS)

Perceived organizational support was measured using the 13-item Perceived Organizational Support Scale (POSS) originally developed by Eisenberger et al. (19) and later revised by Zuo et al. (20). The scale comprises two dimensions: emotional support and instrumental support. Each item is rated on a 5-point Likert scale ranging from 1 (“strongly disagree”) to 5 (“strongly agree”), with total scores ranging from 13 to 65. Higher scores indicate stronger perceived organizational support. The reported Cronbach's alpha of the scale was 0.920, and Cronbach's alpha in the present study was 0.944.

#### 2.3.3 The Decent Work Perception Scale (DWPS)

The 15-item Decent Work Perception Scale (DWPS) developed by Qing et al. (21) was used to assess decent work perception. It comprises three dimensions: respect and recognition, coexistence and inclusion, and confidence and fulfillment. Items are rated on a 5-point Likert scale, yielding total scores from 15 to 75, with higher scores representing higher levels of decent work perception. Cronbach's alpha was 0.925 for the original scale and 0.919 in the present study.

#### 2.3.4 The Job Embeddedness Scale (JES)

Job embeddedness was measured using the 18-item Job Embeddedness Scale (JES) developed by Guo et al. (22). The scale includes three dimensions: professional identity, peer connection, and professional value. Each item is scored on a 5-point Likert scale ranging from 1 (“completely disagree”) to 5 (“completely agree”), with total scores ranging from 18 to 90. Higher scores indicate greater job embeddedness. Cronbach's alpha of the scale was 0.917, and Cronbach's alpha in the current study was 0.955.

#### 2.3.5 The Nurse Safety Behavior Scale (NSBQ)

Nurses' safety behavior was assessed using the 12-item Nurse Safety Behavior Scale (NSBQ) developed by Shih et al. (23) and later translated and adapted into Chinese by Rong (24). This is a unidimensional scale. Each item is rated on a 5-point Likert scale ranging from 1 (“never”) to 5 (“always”), with total scores ranging from 12 to 60. Higher scores indicate better safety behavior. The Cronbach's alpha of the scale ranged from 0.898 to 0.915, and Cronbach's alpha in the present study was 0.934.

### 2.4 Data collection

Data was collected using an electronic questionnaire developed by two trained researchers through the Questionnaire Star platform. Before data collection, permission and support were obtained from the administrators of the participating hospitals. The head nurses of each department then distributed the questionnaire link and QR code to nurses through departmental workgroup chats.

Participation was voluntary. An introductory statement was provided at the beginning of the questionnaire to explain the purpose and content of the study, ensuring that participants completed the survey after fully understanding the study information. To enhance data quality, all returned questionnaires were reviewed by two researchers. Questionnaires with inconsistent personal information, completion times of less than 180 s, or patterned and uniform responses were considered invalid and excluded from analysis.

### 2.5 Data analysis

Data were analyzed using SPSS 26.0 and AMOS 21.0 (IBM Corporation, Armonk, USA). Continuous variables were summarized as means and standard deviations, and categorical variables as frequencies and percentages. Given the non-normal distribution of the data, Spearman's rank correlation analysis was used to examine correlations among the main study variables, and Mann–Whitney *U* or Kruskal–Wallis *H* tests were used to compare nurses' safety behavior across demographic and professional characteristics. Multiple linear regression was performed to identify factors associated with nurses' safety behavior, with multicollinearity assessed using variance inflation factors. Common method bias was evaluated using Harman's single-factor test according to Pei et al. (25). Structural equation modeling with bootstrap resampling was conducted to examine the direct and indirect effects of perceived organizational support on nurses' safety behavior through decent work perception and job embeddedness. A two-tailed *p-*value of < 0.05 was considered statistically significant.

### 2.6 Ethical considerations

This study was approved by the hospital ethics committee (Approval No. 2024YJSLL-06). Participation was entirely voluntary, and informed consent was obtained from all participants before questionnaire completion. The survey was completed anonymously, and confidentiality of participants' information was assured throughout the study.

## 3 Results

### 3.1 Participant inclusion and sample characteristics

A total of 795 questionnaires were returned. Of these, 42 were excluded because of inconsistent personal information, such as inconsistencies between age and professional title (*n* = 4), completion times of less than 180 s (*n* = 26), or patterned or uniform responses indicating invalid completion (*n* = 12). The final sample comprised 753 nurses, yielding an effective response rate of 94.7%.

Most participants were female (89.4%). The most common age groups were 20–30 years and 31–40 years, accounting for 43.7% and 38.0% of the sample, respectively. Most participants held a bachelor's degree (79.02%) and were married (62.9%). Regarding professional characteristics, nurses with the professional title of senior nurse or above accounted for a substantial proportion of the sample (74.9%), and the distribution of years of work experience was relatively balanced. In addition, 41.0% of participants had teaching responsibilities, and most had participated in standardized training (75.8%).

Table 1 presents the non-parametric test results of nurses' safety behavior scores across different demographic characteristics. Significant differences in nurses' safety behavior scores were found across professional title groups (*H* = 10.180, *P* = 0.017), teaching responsibilities (*Z* = −11.630, *P* <0.001), and participation in standardized training (*Z* = −6.217, *P* <0.001). No statistically significant differences were found according to sex, age, educational level, marital status, or years of work experience. Dunn's *post-hoc* pairwise comparisons with Bonferroni correction showed no statistically significant pairwise differences between professional title groups (see Supplementary Table 1).

**Table 1:** Non-parametric tests of nurses' safety behavior scores across demographic characteristics (*n* = 753).

Variable	*n* (%)	Safety behavior score M (P_25_, P_75_)	Mean rank	Test statistic	*P*
Sex				*Z* = −0.858	0.391
Male	80 (10.6)	56.50 (53.25, 58.00)	357.44		
Female	673 (89.4)	57.00 (53.50, 58.00)	379.32		
Age, years				*H* = 3.038	0.386
20–30	329 (43.7)	57.00 (53.00, 58.00)	377.93		
31–40	286 (38.0)	57.00 (53.00, 58.00)	365.14		
41–50	116 (15.4)	57.00 (54.00, 58.00)	405.97		
>50	22 (2.9)	56.50 (53.75, 58.00)	364.55		
Educational level				*H* = 1.042	0.594
Junior college or below	150 (19.92)	57.00 (53.00, 58.00)	378.89		
Bachelor's degree	595 (79.02)	57.00 (53.00, 58.00)	375.5		
Master's degree or above	8 (1.1)	56.50 (56.00, 59.00)	453.31		
Marital status				*H* = 0.764	0.682
Married	474 (62.9)	57.00 (54.00, 58.00)	373.15		
Single	263 (35.0)	57.00 (53.00, 58.00)	381.64		
Divorced/widowed	16 (2.1)	57.00 (53.00, 58.75)	414.78		
Professional title				*H* = 10.180	0.017
Nurse	189 (25.1)	57.00 (53.50, 58.00)	394.37		
Senior nurse	293 (38.9)	57.00 (54.00, 58.00)	394.34		
Nurse-in-charge	233 (30.9)	56.00 (52.50, 58.00)	352.64		
Associate chief nurse or above	38 (5.0)	55.00 (53.00, 57.00)	306.29		
Years of work experience				*H* = 3.159	0.368
<5	216 (28.7)	57.00 (53.25, 58.00)	387.73		
5–10	188 (25.0)	57.00 (52.00, 58.00)	356.91		
11–15	160 (21.2)	57.00 (53.00, 58.00)	369.53		
>15	189 (25.1)	57.00 (54.00, 58.00)	391.04		
Teaching responsibilities				*Z* = −11.630	<0.001
Yes	309 (41.0)	58.00 (57.00, 59.00)	486.55		
No	444 (59.0)	55.00 (51.00, 57.00)	300.76		
Participation in standardized training				*Z* = −6.217	<0.001
Yes	571 (75.8)	57.00 (54.00, 58.00)	404.58		
No	182 (24.2)	56.00 (50.00, 57.00)	290.46		

Data are presented as *n* (%) or median (P_25_, P_75_). Mann–Whitney *U* tests were used for comparisons between two groups, and Kruskal–Wallis *H* tests were used for comparisons among three or more groups. *Z-* and *H*-values are reported as test statistics. *P* <0.05 was considered statistically significant.

### 3.2 Descriptive statistics and Spearman's rank correlation analysis

The mean total scores for perceived organizational support, decent work perception, job embeddedness, and nurses' safety behavior were 40.92 ± 9.86, 55.99 ± 7.59, 65.30 ± 11.08, and 55.28 ± 4.22, respectively.

Spearman's rank correlation analysis showed that nurses' safety behavior was significantly and positively correlated with perceived organizational support, decent work perception, and job embeddedness (ρ = 0.445–0.617, *P* <0.01). Significant correlations were also observed among the predictor variables, with perceived organizational support showing a moderate to strong correlation with job embeddedness (ρ = 0.619, *P* <0.01). Multicollinearity diagnostics indicated that all variance inflation factor (VIF) values ranged from 1.015 to 6.355, suggesting no serious multicollinearity (see Supplementary Table 2 and Table 2).

**Table 2:** Correlations among study variables.

Variables	1	2	3	4	5	6	7	8	9	10	11	12
1.Safety behavior	1	–	**–**	**–**	**–**	**–**	**–**	**–**	**–**	**–**	**–**	**–**
2.Perceived organizational support	0.605^**^	1	**–**	**–**	**–**	**–**	**–**	**–**	**–**	**–**	**–**	**–**
3.Emotional support	0.598^**^	0.980^**^	1	–	–	–	–	–	–	–	–	–
4.Instrumental support	0.462^**^	0.782^**^	0.660^**^	1	–	–	–	–	–	–	–	–
5.Decent work perception	0.531^**^	0.488^**^	0.487^**^	0.335^**^	1	–	–	–	–	–	–	–
6.Respect	0.503^**^	0.489^**^	0.482^**^	0.352^**^	0.921^**^	1	–	–	–	–	–	–
7.Inclusion	0.496^**^	0.433^**^	0.438^**^	0.277^**^	0.925^**^	0.782^**^	1	–	–	–	–	–
8.Confidence	0.445^**^	0.398^**^	0.397^**^	0.285^**^	0.878^**^	0.714^**^	0.751^**^	1	–	–	–	–
9.Job embeddedness	0.617^**^	0.619^**^	0.611^**^	0.462^**^	0.659^**^	0.613^**^	0.600^**^	0.575^**^	1	–	–	–
10.Professional identity	0.594^**^	0.602^**^	0.591^**^	0.468^**^	0.644^**^	0.594^**^	0.587^**^	0.569^**^	0.946^**^	1	–	–
11.Peer connection	0.600^**^	0.577^**^	0.567^**^	0.439^**^	0.615^**^	0.569^**^	0.568^**^	0.533^**^	0.947^**^	0.876^**^	1	–
12.Professional value	0.553^**^	0.552^**^	0.550^**^	0.383^**^	0.591^**^	0.555^**^	0.536^**^	0.511^**^	0.902^**^	0.780^**^	0.792^**^	1

ρ = Spearman's rank correlation coefficient. ^**^P <0.01.

### 3.3 Mediating effect analysis

**Figure 1:**
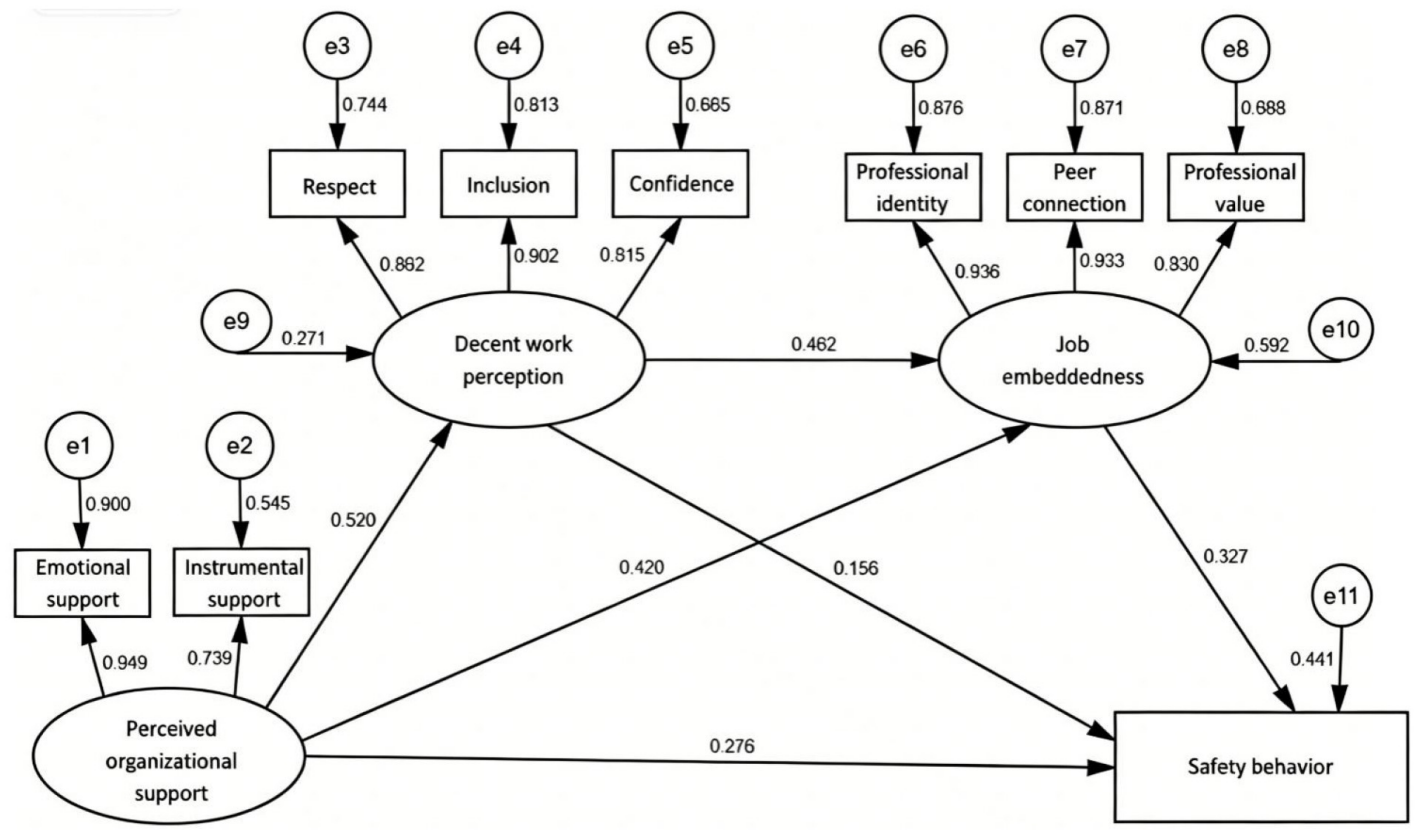
Structural equation model of the mediating effects of decent work perception and job embeddedness on the relationship between perceived organizational support and nurses' safety behavior.

A structural equation model was constructed in AMOS to examine the mediating roles of decent work perception and job embeddedness in the association between perceived organizational support and nurses' safety behavior (Figure 1). The model showed a good fit to the data (26), with χ^2^/df = 1.791, GFI = 0.989, AGFI = 0.977, CFI = 0.996, TLI = 0.994, and RMSEA = 0.032.

As shown in Figure 1, perceived organizational support, decent work perception, and job embeddedness all had significant positive direct effects on nurses' safety behavior (β = 0.276, β = 0.156, and β = 0.327, respectively; *P* <0.001). In addition, perceived organizational support had significant positive effects on decent work perception (β = 0.520) and job embeddedness (β = 0.420), while decent work perception was positively associated with job embeddedness (β = 0.462).

The mediating effects were further tested using bootstrap resampling with 5,000 samples. Mediation was considered significant when the 95% bootstrap confidence interval did not include zero. The total effect of perceived organizational support on nurses' safety behavior was 0.573, of which the direct effect was 0.276, accounting for 48.17% of the total effect. The total indirect effect was 0.297, accounting for 51.83% of the total effect. Specifically, the indirect effect through decent work perception alone was 0.081 (Boot SE = 0.023, 95% CI: 0.036–0.128), accounting for 14.14% of the total effect. The indirect effect through job embeddedness alone was 0.137 (Boot SE = 0.025, 95% CI: 0.092–0.189), accounting for 23.90% of the total effect. The sequential indirect effect through decent work perception and job embeddedness was 0.079 (Boot SE = 0.015, 95% CI: 0.054–0.113), accounting for 13.79% of the total effect (Table 3). These findings indicate that decent work perception and job embeddedness mediated the relationship between perceived organizational support and nurses' safety behavior both independently and sequentially.

**Table 3:** Bootstrap test of the mediating effects of decent work perception and job embeddedness on the relationship between perceived organizational support and nurses' safety behavior.

Effect type	Effect value	Boot SE	Bootstrap 95% CI	Proportion of total effect (%)
Total effect	0.573	0.022	0.528–0.613	–
Direct effect	0.276	0.037	0.203–0.350	48.17
Total indirect effect	0.297	0.027	0.246–0.351	51.83
POS → DWP → NSB	0.081	0.023	0.036–0.128	14.14
POS → JE → NSB	0.137	0.025	0.092–0.189	23.9
POS → DWP → JE → NSB	0.079	0.015	0.054–0.113	13.79

POS, perceived organizational support; DWP, decent work perception; JE, job embeddedness; NSB, nurses' safety behavior; SE, standard error; CI, confidence interval.

## 4 Discussion

### 4.1 Nurses' safety behavior

In this study, the mean total score of nurses' safety behavior was 55.28 ± 4.22, indicating a moderately high level of safety behavior. Significant differences were observed across professional title groups, teaching responsibilities, and participation in standardized training. Nurses with teaching responsibilities and those who had completed standardized training demonstrated higher safety behavior scores, suggesting that clinical teaching roles and structured training may be associated with improved safety performance.

Differences across professional title groups may be related to variations in clinical experience, role expectations, and familiarity with safety procedures. Nurses with higher professional titles generally possess greater clinical experience and professional competence, which may facilitate more standardized safety practices (27). However, after controlling for perceived organizational support, decent work perception, job embeddedness, and other variables, professional title was no longer an independent predictor of safety behavior, suggesting that its effect may be mediated by work-related psychological and organizational factors. Sex, age, educational level, marital status, and years of work experience were not significantly associated with nurses' safety behavior in either univariate or multivariate analyses. Although previous evidence has suggested that demographic characteristics may influence safety behavior through professional role adaptation and work–family balance (28, 29), the relatively homogeneous sample in this study (predominantly female, young-to-middle-aged, and bachelor's degree educated) may have limited between-group variability.

Teaching responsibilities and participation in standardized training were associated with nurses' safety behavior. Nurses with teaching responsibilities may reinforce safety standards through repeated demonstration of clinical procedures, supervision of junior staff, and correction of unsafe practices (30). Standardized training can facilitate the systematic acquisition of knowledge related to clinical safety procedures, risk prevention, and emergency management (31). These findings may help explain the moderately high level of safety behavior observed in this study. Compared with a previous study conducted in Beijing (32), the safety behavior score in the present study was relatively lower. This difference may be attributed to variations in hospital management models, safety culture, organizational support, training resources, and nurses' job embeddedness across regions. Accordingly, nursing managers should not rely solely on demographic characteristics such as age or professional title when designing interventions. Instead, targeted safety training should be strengthened for nurses without teaching responsibilities or standardized training experience, while the exemplary role of senior nurses may be leveraged to improve safety behavior. Cross-regional sharing of safety management experience may further enhance the implementation quality of nurses' safety practices.

### 4.2 The mediating role of decent work perception in the relationship between perceived organizational support and nurses' safety behavior

Decent work perception partially mediated the relationship between perceived organizational support and nurses' safety behavior, with an indirect effect of 0.081, accounting for 14.14% of the total effect. This finding indicates that decent work perception serves as an important psychological mechanism linking perceived organizational support to nurses' safety behavior. This result can be interpreted from conservation of resources theory, which suggests that individuals strive to obtain and preserve valuable psychological resources when facing work demands (33). In high-demand clinical environments, decent work perception may help nurses maintain psychological resources, professional motivation, and work responsibility, thereby reducing unsafe practices. Empirical evidence supports this interpretation. Xue et al. (34) reported that decent work perception reflects nurses' evaluation of their working environment and is closely related to the nursing practice context. Su and Chan (35) further found that decent work perception is positively associated with occupational wellbeing and work engagement. Based on these findings, decent work perception may function as a key cognitive link through which perceived organizational support translates into improved safety behavior. From a managerial perspective, strengthening professional recognition, expanding career development opportunities, and enhancing the perceived value of nursing work may help improve nurses' decent work perception and, in turn, promote safer clinical practice.

### 4.3 The mediating role of job embeddedness in the relationship between perceived organizational support and nurses' safety behavior

Job embeddedness mediated the relationship between perceived organizational support and nurses' safety behavior, with an indirect effect of 0.137, accounting for 23.90% of the total effect. This represents the strongest specific indirect effect in the model. This finding suggests that perceived organizational support may enhance nurses' safety behavior by strengthening their attachment to their job, colleagues, and organization. When nurses perceive that their organization values their contributions, supports their professional development, and provides adequate resources, they are more likely to develop a stronger sense of organizational belonging and professional identity, which in turn promotes stable and safety-oriented clinical behavior. Empirical evidence supports this interpretation. Al-Hamdan and Bani Issa (36) reported that perceived organizational support is positively associated with work engagement, indicating that organizational resources can facilitate positive work-related attitudes. In contrast, the demanding and high-risk nature of nursing work has been shown to weaken job embeddedness (37). Building on these findings, the present study further demonstrates that job embeddedness serves as a key mediating mechanism linking perceived organizational support to nurses' safety behavior. From an applied perspective, interventions should not only focus on technical safety training but also strengthen nurses' organizational belonging, professional identity, and collegial support to enhance safety-related outcomes.

### 4.4 The sequential mediating roles of decent work perception and job embeddedness in the relationship between perceived organizational support and nurses' safety behavior

This study found that decent work perception and job embeddedness sequentially mediated the relationship between perceived organizational support and nurses' safety behavior, with an indirect effect of 0.079, accounting for 13.79% of the total effect. This finding suggests that perceived organizational support may first enhance nurses' perception of work dignity and value, which subsequently strengthens their job embeddedness and ultimately promotes safety behavior. This sequential pathway provides a more comprehensive explanation of how organizational resources are translated into safety-related outcomes. Perceived organizational support represents an external organizational resource, whereas decent work perception reflects nurses' cognitive and emotional evaluation of the dignity and value of their work. In turn, job embeddedness reflects nurses' psychological attachment to their job, colleagues, and organization. Empirical evidence provides indirect support for this mechanism. Cao et al. (38) reported that organizational support can enhance nurses' subjective wellbeing, which may further improve their perception of decent work. Other studies have shown that decent work perception is positively associated with work engagement and professional growth (39), which may strengthen nurses' organizational attachment. Overall, these findings are consistent with a sequential process in which organizational support enhances decent work perception, which in turn strengthens job embeddedness and ultimately promotes nurses' safety behavior. Such improvements in nurses' safety behavior may further contribute to better patient safety, nursing care quality, and hospital performance (40).

### 4.5 Clinical implications

The findings highlight that clinical teaching, standardized training, perceived organizational support, decent work perception, and job embeddedness are key factors associated with nurses' safety behavior. At the practice level, hospitals should strengthen structured clinical teaching and standardized training systems, particularly for newly recruited and less experienced nurses. Training should focus on standardized nursing procedures, risk identification, adverse event prevention, and emergency response. Experienced nurses with strong safety performance may serve as clinical mentors to reinforce safety behavior through demonstration, supervision, and feedback. Nursing managers should also enhance perceived organizational support by ensuring timely feedback, adequate resources, supportive communication, and recognition of nurses' contributions. Public recognition of exemplary nursing practice may further strengthen nurses' professional value and social identity (41). To improve decent work perception, hospitals may expand professional development opportunities through specialist nurse-led clinics, “Internet + nursing service” models (42), and, where feasible, extended nursing roles such as nurse prescribing (43). These strategies may enhance nurses' sense of dignity, autonomy, and professional value. Finally, strengthening job embeddedness may help sustain safety behavior over time. This may be achieved by supporting individualized career development, improving promotion pathways, providing opportunities for research and clinical practice (44), and promoting team-building, cross-department collaboration, and peer support (45). These measures may enhance professional identity, organizational belonging, and perceived professional value, thereby supporting long-term improvements in nurses' safety behavior and workforce stability (46).

### 4.6 Limitations

This study has several limitations. First, the cross-sectional design limits causal inference, and longitudinal studies are needed to confirm the temporal relationships among variables. Second, self-reported data may introduce reporting bias. Third, although data were collected between October and December 2024 and the manuscript was submitted in April 2026, temporal changes in hospital management, staffing, and safety culture during this period may affect the applicability of the findings. Fourth, although nurses with unstable clinical roles were excluded, no stratified or sensitivity analyses were conducted according to years of clinical experience. Fifth, although multicollinearity diagnostics indicated no severe multicollinearity, perceived organizational support and job embeddedness were moderately to strongly correlated, which should be considered in interpretation of the findings. Finally, convenience sampling from four tertiary hospitals in one region of China may limit the generalizability of the results. Future studies should include more diverse populations and incorporate additional organizational and contextual variables.

## 5 Conclusions

Perceived organizational support was positively associated with nurses' safety behavior, and decent work perception and job embeddedness played independent and sequential mediating roles in this relationship. These findings suggest that interventions to improve nurses' safety behavior should integrate organizational support with efforts to enhance nurses' perceived work value and job embeddedness, thereby promoting safer nursing practice and better care quality.

## Data Availability

The original contributions presented in the study are included in the article/Supplementary material, further inquiries can be directed to the corresponding author.
