# Supplementary material for: The impact of perceived organizational support on nurses' safety behavior: the sequential mediating roles of decent work perception and job embeddedness
Source: Front Public Health. 2026 Jul 28;14:1868271. doi: 10.3389/fpubh.2026.1868271 (PMC13457307; doi:10.3389/fpubh.2026.1868271)
Supplement: Supplementary file 1 [file Data_Sheet_1.pdf]

Supplementary Table 1 Dunn's pairwise comparisons of nurses' safety behavior scores across professional title groups after Bonferroni correction

Comparison group	Standardized Z	Adjusted P	Between-group difference
Associate chief nurse or above vs nurse-in-charge	1.229	1	Not statistically significant
Associate chief nurse or above vs senior nurse	2.368	0.107	Not statistically significant
Associate chief nurse or above vs nurse	2.297	0.13	Not statistically significant
Nurse-in-charge vs senior nurse	2.203	0.166	Not statistically significant
Nurse-in-charge vs nurse	1.977	0.288	Not statistically significant
Senior nurse vs nurse	0.001	1	Not statistically significant

Note. Dunn's post hoc pairwise comparisons were performed with Bonferroni correction for multiple comparisons. Adjusted $P < 0.05$ was considered statistically significant.

Supplementary Table 2. Multiple Linear Regression Analysis of Predictors for Nurses' Safety Behavior Scores and Multicollinearity Diagnosis of Three Core Latent Variables (n = 753)

Variable	Unstandardized coefficient B	SE	Standardized coefficient β	t	P	VIF
Demographic characteristics and sub-dimension items of latent constructs						
Constant	41.539	1.655	—	25.093	<0.001	—
Sex	-0.168	0.367	-0.012	-0.458	0.647	1.015
Age	-0.007	0.036	-0.013	-0.192	0.848	6.344
Educational level	0.431	0.294	0.043	1.468	0.142	1.194
Professional title	0.290	0.205	0.059	1.411	0.159	2.469
Years of work experience	0.019	0.246	0.005	0.078	0.938	6.355
Teaching responsibilities	-1.469	0.272	-0.171	-5.408	<0.001	1.416
Participation in standardized training	-0.708	0.300	-0.072	-2.363	0.018	1.306
Marital status	-0.094	0.255	-0.012	-0.368	0.713	1.453
Emotional support	0.110	0.022	0.212	5.020	<0.001	2.515
Instrumental support	0.050	0.070	0.028	0.725	0.468	2.039
Respect	0.063	0.059	0.049	1.054	0.292	3.003
Inclusion	0.213	0.077	0.135	2.777	0.006	3.335
Confidence	-0.063	0.074	-0.036	-0.853	0.394	2.505
Professional identity	0.093	0.054	0.103	1.711	0.087	5.109
Peer connection	0.249	0.068	0.217	3.669	<0.001	4.969
Professional value	-0.009	0.055	-0.007	-0.158	0.875	2.931
Three core latent constructs (total scale scores)						
Constant	37.356	0.895	—	41.745	<0.001	—
Perceived organizational support	0.116	0.015	0.270	7.609	<0.001	1.630
Decent work perception	0.091	0.020	0.163	4.515	<0.001	1.685
Job embeddedness	0.124	0.015	0.326	8.113	<0.001	2.089

Note. SE = standard error; VIF = variance inflation factor.
